# Hypothermia drives fatty acid transporter 1 upregulation and lipid accumulation in renal tubules: evidence from forensic cases

**DOI:** 10.1007/s00414-025-03550-x

**Published:** 2025-06-19

**Authors:** Kie Horioka, Hiroki Tanaka, Akira Hayakawa, Yoichiro Takahashi, Henrik Druid, Lasse Pakanen, Katja Porvari

**Affiliations:** 1 Department of Forensic Medicine, Research Unit of Internal Medicine, Medical Research Center Oulu, University of Oulu, Aapistie 5A, Oulu, 90220 Finland; 2 Department of Legal Medicine, Institute of Medicine, University of Tsukuba, Tsukuba, Japan; 3 Department of Oncology-Pathology, Karolinska Institutet, Stockholm, Sweden; 4 Laboratory of Molecular Diagnostics and Therapeutics, National Institutes of Biomedical Innovation, Health and Nutrition (NIBN), Ibaraki, Japan; 5 Department of Forensic Sciences, Akita University Graduate School of Medicine, Akita, Japan; 6 Forensic Medicine Unit, Finnish Institute for Health and Welfare (THL), Oulu, Finland

**Keywords:** Hypothermia, Renal tubular cells, Lipid droplets, FATP1, Histopathological examination

## Abstract

Forensic pathology lacks generally accepted markers for hypothermia, relying instead on various physiological responses to low temperatures as postmortem diagnostic indicators. We have demonstrated that fatty acid transporter 1 (FATP1) is upregulated in renal tubules of a mouse hypothermia model, causing lipid accumulation. This study aims to determine if a similar phenomenon occurs in cases of human hypothermia and evaluate its potential as a diagnostic tool. Blood and renal tissue samples were collected from 17 hypothermia cases and 23 non-hypothermia cases. Free fatty acid (FFA) and triglyceride levels were quantified in both blood and renal tissue, while mRNA and protein were extracted from renal tissue to assess FATP1 expression. Oil Red O staining revealed lipid-positive droplets in renal tubular cells in hypothermia cases. Both FFA and triglyceride levels were significantly elevated in the blood and renal tissue samples of hypothermia cases. In cultured human renal tubular cells, low temperature upregulated FATP1 expression and FFA uptake, while FATP1 inhibition reduced FFA uptake. FATP1 mRNA and protein expression were significantly increased in hypothermic renal tissue compared with those in controls. Additionally, this expression positively correlated with FFA content in hypothermic renal tissue. These findings suggest that FATP1-dependent lipid accumulation in renal tubules is a conserved response in both animal models and human hypothermia cases, highlighting its potential as a diagnostic marker in forensic investigations of fatal hypothermia.

## Introduction

Forensic pathology currently lacks specific diagnostic markers for hypothermia of the deceased, relying instead on indirect assessments of physiological responses to low temperatures or exclusion-based diagnoses to rule out other causes of death. Previous studies reported postmortem findings of hypothermia, for example, Wischnewsky spots in the gastric mucosa and differences in the color of left and right ventricular blood [1]. One of the main features of fatal hypothermia is the presence of lipid droplets in renal tubules [2, 3]. The degree of lipid degeneration in renal tubules can be a useful indicator for diagnosing death due to hypothermia, having a diagnostic sensitivity equivalent to that of Wischnewsky spots [2]. Also, free fatty acid (FFA) concentrations of saturated and unsaturated fatty acids in blood were significantly raised in fatal hypothermia, regardless of blood alcohol concentration [4, 5]. However, the mechanisms and biological significance of lipid dynamics during hypothermia have not been fully elucidated.

Generally, renal tubular cells mainly use fatty acid oxidation as the energy source [6]. FFA are mainly transported into tubular cells by cluster of differentiation 36 (CD36) and the fatty acid transporter protein (FATP/Slc27) family, leading to the accumulation of triglyceride-enriched lipid droplets (LDs) [7]. Then, fatty acids are transported to the mitochondria for oxidation. It is also known that the gene SLC27A1, mRNA encoding the FATP1 protein plays a key role in lipid metabolism to promote β-oxidation in renal tissue and that PPARα regulates its expression [8]. In our previous study using a mouse model of hypothermia [9], we reported a notable phenomenon: lipid droplet accumulation in the renal tubules that was dependent on the expression of FATP1 and several fatty acid metabolism factors. If the mechanisms by which these changes occur are common to humans and rodents, this could provide new insights into lipid metabolism under hypothermic conditions and may significantly advance our understanding of the renal response to hypothermia in humans.

Improved techniques has enabled the use of RNA in postmortem research [10–13]. Although forensic RNA analysis remains limited, it holds promise for revealing biological reactions before death by offering insights similar to RNA analyses of living tissues. We previously reported that expression of AREG mRNA (a member of the epidermal growth factor family) is elevated in the hearts of hypothermia autopsy cases, making it a specific biomarker for hypothermia diagnosis independent of stress hormones [14]. This mRNA analysis also effectively revealed antemortem gene expression patterns connected specifically to cardiovascular diseases [14]. Elevated AREG mRNA appears to be a unique response to hypothermia rather than a general acute stress marker, suggesting that cold exposure triggers distinct biological responses before death. Therefore, analysis of mRNA expression patterns in tissue samples from forensic autopsies of hypothermia cases may contribute to our understanding of hypothermia. Additionally, the forensic diagnosis of hypothermia becomes more accurate when based on a combination of various low-temperature-dependent gene expression features, along with already established macroscopic and histologic findings obtained from affected organs. This multi-faceted approach enhances the reliability of the diagnosis.

In this study, we investigated the relationship between hypothermia induced FFA levels and select lipid metabolism-related factors in human autopsy samples and renal proximal tubular epithelial cells.

## Materials and methods

### Autopsy samples

Study cases were selected based on autopsy reports and cause of death (Table 1). In total, 23 control cases [7 coronary artery disease cases, 5 intoxications (drugs and medication or ethanol), 5 suffocations (include hanging), 1 chronic drug abuse subject with acute pneumonia, 1 chronic alcohol use subject, 1 case with multiple rib fractures, and 1 case with ruptured aneurysm of thoracic aorta] and 17 hypothermia cases (including 10 cases of hypothermia as a contributory cause of death) were included. The diagnosis of hypothermia was based on multiple criteria: documented cold exposure (including environmental circumstances, clothing, signs of paradoxical undressing), autopsy findings suggestive of cold exposure and hypothermia (such as external signs and the presence of Wischnewsky’s spots in the gastric mucosa), toxicological and biochemical investigations (including urine catecholamine levels, concentration of vitreous glucose, lactate and β-hydroxybutyrate in blood), and the exclusion of other potential causes of death [1, 15]. The bodies were stored at 4 °C until autopsy. Kidney tissue samples were collected during autopsy, immediately frozen at -80 °C and stored until extraction of RNA and protein. Permission to use cadaveric tissue samples and cause-of-death investigation data was granted by Finnish Institute for Health and Welfare (Dnro THL/2542/5.05.00/2021). The Northern Ostrobothnia Hospital Research Ethics Committee approved the original hypothermia study plan (31/2007). Tissue samples were collected for research purposes in medicolegal autopsies performed in the Forensic Medicine Unit, Finnish Institute for Health and Welfare, Oulu, Finland.

**Table 1:** Case information

Cause of death	Age	Sex	PMI
	(year; mean + SE)	(*n*; males/females)	(days; median, range)
Control (*n* = 23)	46.5 + 5.0	15/8	7.4 (2–16)
Hypothermia (*n* = 17)	57.1 + 4.8	10/7	9.4 (5–27)
All groups (*n* = 40)	51.2 + 3.6	25/15	8.3 (2–27)

### Biochemical analysis

Blood and tissue FFA and triglyceride (TG) concentrations were measured by enzymatic chemical reaction (LabAssay™ Non-Esterified Fatty Acid and LabAssay™ Triglyceride, FUJIFILM, Osaka, Japan). Serum was obtained from blood collected from autopsies by centrifugation at 1000 × g for 10 min. To analyze the contents of FFA and TG in renal tissue, cortical portions of the kidney were homogenized in 0.5% NP-40 in phosphate-buffered saline, and the centrifuged supernatant was used as a sample.

### Histopathological analysis

Oil Red O (ORO) staining was performed on frozen sections to visualize lipid droplets in renal tubular cells. Tissue specimens were initially fixed in 10% solution of formaldehyde, then embedded in an OCT compound (SAKURA, Tokyo, Japan) and frozen using hexane coolant. Kidney samples were immersed in 10% solution of formaldehyde overnight, followed by immersion in 30% sucrose. The tissues were then in embedded in an OCT compound (SAKURA), frozen using FlashFREEZE system (Milestone, Valbrembo, Italy), and sectioned at a thickness of 5 μm using a Leica CM3050 S cryostat (Leica, Deer Park, IL, USA). Lipid droplets in renal tubular cells were analyzed by ORO application. ORO-positive cells were graded on a three-tier scale at high magnification, as described by Preuss. et al. [2].: Negative (no lipid droplets), Low (slight presence of lipid droplets in random renal tubules per high-power field), and High (moderate to severe lipid droplet presence, with more than 50% of renal tubules showing positive lipid droplets per high-power field). Formalin-fixed, paraffin-embedded tissue samples were prepared in accordance with a standard protocol. For immunohistochemical analysis, 3-µm-thick paraffin sections were sequentially treated before application with the primary antibody as follows: deparaffinization, rehydration, endogenous peroxidase quenching, and antigen retrieval with 0.1 M sodium citrate buffer in a microwave. Sections were then incubated with a rabbit polyclonal FATP1 primary antibody (Novus Biologicals, Littleton, CO, USA), followed by detection with a horseradish peroxidase (HRP)-conjugated secondary rabbit IgG antibody (Vector Labs, Burlingame, CA, USA). FATP1 expression in renal tubular cells was graded by a similar grading system of immunohistochemistry staining with HSP70, as reported by Preuss et al. [2]. We graded as follows in 20 high-power fields magnification: 0, no staining; 1+, mild but clear cytoplasmic positive staining; 2+, moderate cytoplasmic positive staining; and 3+, strong and complete cytoplasmic positive staining.

### Cell culture

Human renal proximal tubular epithelial cells (HK2) were purchased from ATCC (Manassas, VA, USA). These cells were cultured in Dulbecco’s Modified Eagle Medium supplemented with 10% (vol/vol) fetal bovine serum and penicillin-streptomycin, in a humidified atmosphere containing 5% CO_2_. For low-temperature stimulation, cells were seeded into culture plates containing culture medium and subsequently treated with growth medium containing 0.1 mM palmitic acid (PA) and/or a FATP1 inhibitor (FATP1-in-1; TargetMol, Wellesley Hills, MA, USA). Cells were then incubated at either 37 °C (Cont) or 28 °C (Hypo) for 15 h. BODIPY staining was performed to assess FFA uptake under low-temperature conditions. After formalin fixation, cells were stained with BODIPY (Thermo Fisher Scientific, Waltham, MA, USA), observed under a microscope, and quantified using a fluorescence plate reader.

### Real-time quantitative polymerase chain reaction (qRT-PCR)

Total RNA was isolated from cultured HK2 cells and kidney samples with an miRNeasy Mini Kit (QIAGEN, Hilden, Germany) using an automated QIAcube sample preparation instrument (QIAGEN) according to the manufacturer’s protocol. One-step real-time RT-PCR with SYBR Green I detection was used. RT-PCR was performed using the Rotor-Gene Q system (QIAGEN) for human *SLC27A1*, *PPARA*, *CD36*, and *ACTB* mRNA as an internal control. Each threshold cycle was obtained, and the double-delta threshold cycle method was used to calculate expression values. Primer sequences were as follows for human *SLC27A1*, 5ʹ-CGTGCTAGTGATGGATGAGC-3ʹ and 5ʹ-GCCTCGTCTTCTGGATCTTG-3ʹ; *PPARA*, 5ʹ-CTGGAAGCTTTGGCTTTACG-3ʹ and 5ʹ-CAATGCTCCACTGGGAGACT-3ʹ; *CD36*, 5ʹ-GGCTGCAGGTCAACCTATTG-3ʹ and 5ʹ-GCAACAAACATCACCACACC-3ʹ; and *ACTB*, 5ʹ-GGCATCCTCACCCTGAAGTA-3ʹ and 5ʹ-GGGGTGTTGAAGGTCTCAAA-3ʹ. RT-PCR was carried out as follows: after the reverse-transcription reaction (50 °C, 30 min), amplification was performed with one cycle of denaturation (95 °C, 15 min) followed by 40 cycles of three-stage PCR (95 °C, 30 s; 60 °C, 30 s; and 72 °C, 1 min). Each cycle threshold (CT) value was obtained and the 2^− ΔΔCT^ method was used to calculate relative expression values.

### Western blot analysis

We extracted protein from cultured HK2 cells and the cortical portion of kidneys with RIPA buffer. Kidney samples were homogenized using a bead crusher, and the supernatants were isolated after centrifugation of 1000 ×g for 5 min. Protein samples were then separated in polyacrylamide-sodium dodecyl sulfate gels and electro-transferred to nitrocellulose membranes. After blocking using 5% skim milk, the membranes were probed with an anti-FATP1 rabbit monoclonal antibody (Mybiosouce, San Diego, CA, USA) and anti β-actin mouse monoclonal antibody (BD Biosciences, Franklin Lakes, NJ, USA). The membranes were then incubated with the respective HRP-conjugated anti-rabbit or anti-mouse IgG secondary antibodies (R&D Systems, Minneapolis, MN, USA). Antibody binding was visualized using the SuperSignal West Pico Chemiluminescent Substrate (Thermo Fisher Scientific). β-actin was used as an internal control to normalize the level of target protein. Quantification was performed by densitometry analysis using ImageJ (National Institutes of Health, Bethesda, Maryland, USA).

### Statistics

We used GraphPad Prism (GraphPad Software, San Diego, CA, USA) for all statistical analyses. Differences in experimental values were analyzed via the Mann–Whitney U test or one-way ANOVA followed by the Tukey–Kramer method. To assess correlations, Spearman’s rho test was applied. A *P*-value < 0.05 was considered statistically significant for evaluating differences and correlations in the experimental data.

## Results

### Lipid accumulation in renal proximal tubular tissue

**Fig. 1:**
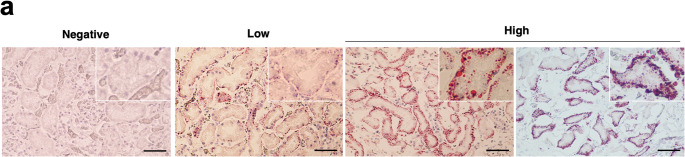
Lipid droplets in renal proximal tubules. Representative Oil Red O staining of human kidney samples from forensic autopsy cases, comparing control and hypothermia groups. Representative examples of each grade are shown. Scale bars: 50 μm

ORO staining was performed on frozen renal tissue samples collected at forensic autopsy that were available for specimen preparation: 23 in the control group and 12 in the hypothermia group. Most of the lipid droplets were located along the proximal tubular basement membrane, and positive ORO results were classified as low grade or high grade (Fig. 1). Two of 23 cases were classified as high grade in the control group, while 9 of 12 cases were classified as high grade in the hypothermia group (Table 2). ORO results clearly differentiated the control group from the hypothermia group, as confirmed by a χ² test (*P* < 0.05).

**Table 2:** Positive rate of oil red O in control and hypothermia cases

	Grade			Positive rate
	Negative	Low	High	(%)
Control (*n* = 23)	21	0	2	9
Hypothermia (*n* = 13)	3	3	7	75

### Biochemical analysis of FFA and TG in blood and renal tissue from hypothermia cases

**Fig. 2:**
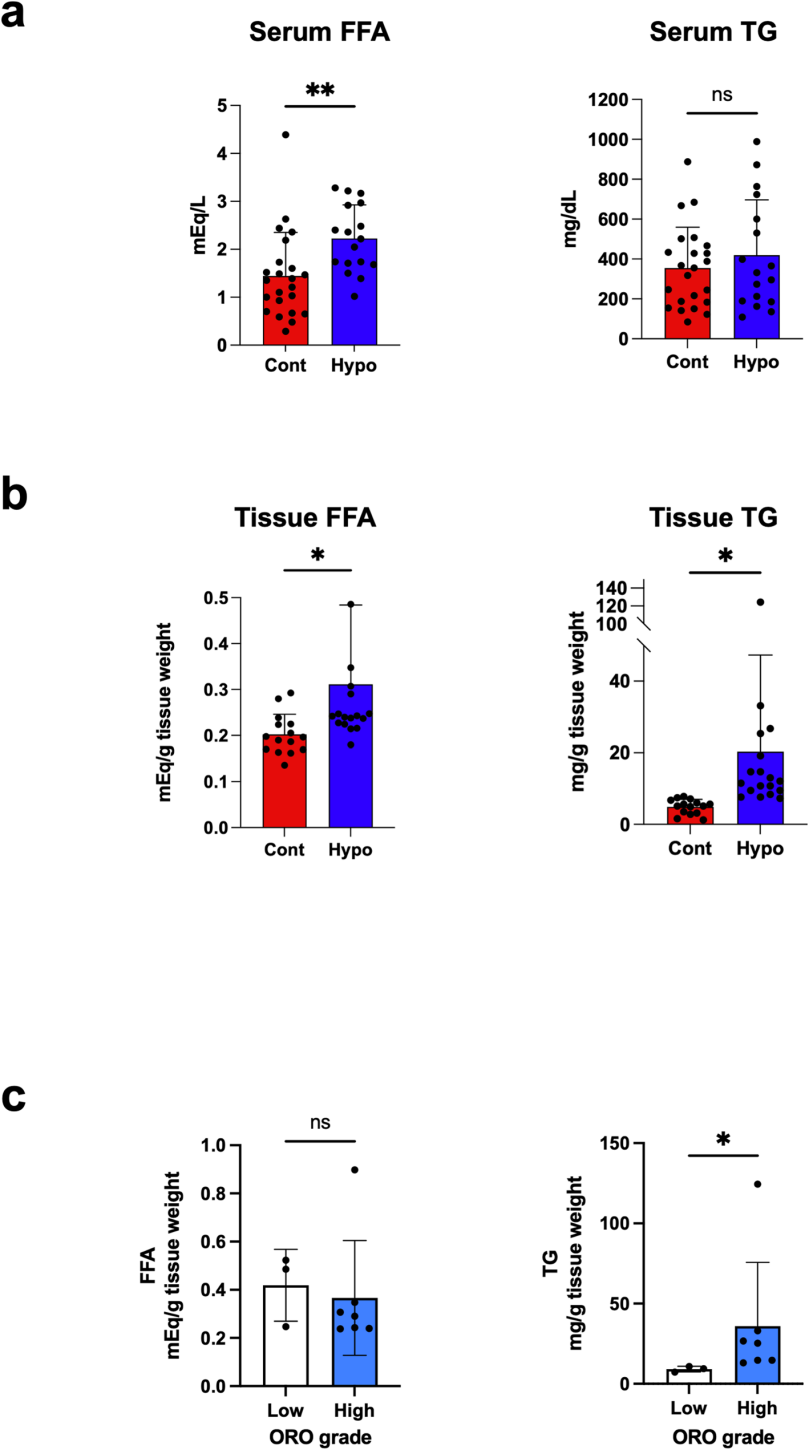
Concentration of lipids in blood and kidney tissue. (**a**, **b**) Total FFA and TG concentrations analyzed by enzymatic method in control and hypothermia cases using serum and homogenized kidney. (**a**) Blood FFA (left) and TG (right), (**b**) Tissue FFA (left) and TG (right) (Cont: *n* = 23, Hypo: *n* = 17). (**c**) FFA (left) and TG (right) content by ORO grade. Cont; control group and Hypo: hypothermia groups. Data were analyzed by the Mann–Whitney U test. All data are represented as mean ± SEM. *P < 0.05, **P < 0.01. FFA, free fatty acid; ORO, Oil Red O; TG, triglyceride

Homogenized kidney tissue and blood samples were analyzed to evaluate changes in FFA and TG in hypothermia. Enzymatic biochemical analysis showed no significant difference in serum TG levels between hypothermia and control groups (Fig. 2a). However, FFA levels were markedly higher in the hypothermia group (Fig. 2a). In addition, elevated FFA and TG contents were detected in renal cortical tissue of the hypothermia group (Fig. 2b). In hypothermia cases, kidney tissue TG contents were higher in the ORO high-grade group compared with those in the ORO low-grade group. However, FFA contents in kidney tissue did not differ between ORO grades (Fig. 2c).

### Gene expression and FFA uptake in human renal tubular cells cultured at low temperature

**Fig. 3:**
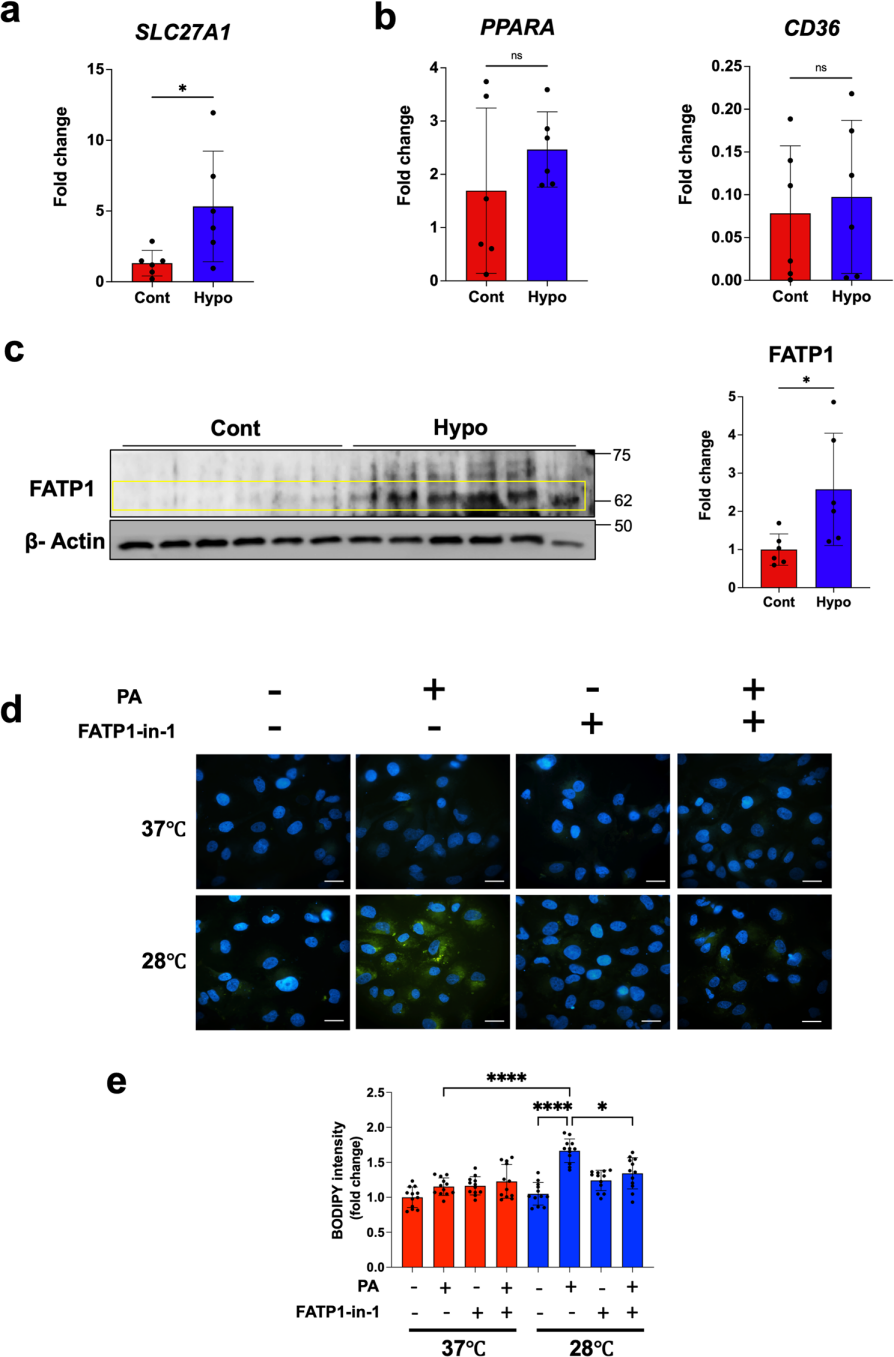
Effect of low temperature on gene expression and lipid accumulation in cultured human proximal tubular cells. (**a**–**c**) Comparison of gene expression in HK2 cells cultured at 37 °C and 28 °C, as analyzed by real-time RT-PCR for (**a**) *SLC27A1* mRNA, (**b**) *PPARA* mRNA, and *CD36* mRNA (*n* = 6 per condition). (**c**) Western blot analysis of FATP1 and β-actin proteins in HK2 cells cultured at 37 °C and 28 °C, with quantitative data derived from western blotting (*n* = 6 per condition). (**d**) Analysis of lipid accumulation in HK2 cells cultured at 37 °C and 28 °C with 0.1 mM palmitic acid (PA) and/or a FATP1 inhibitor. Lipids are visualized using BODIPY (green), with nuclei counterstained with DAPI (blue). Scale bars: 10 μm. (**e**) Quantitative analysis of fluorescence intensity representing lipid accumulation in HK2 cells cultured at 37 °C and 28 °C (*n* = 12 per condition). Data were analyzed using one-way ANOVA followed by the Mann–Whitney U test. All data are presented as mean ± SEM. *P < 0.05, ****P < 0.0001

In our previous analyses of a mouse model of hypothermia and cultured mouse renal tubular cells, we observed low-temperature-dependent transcriptional upregulation of *Slc27a1* mRNA, which encodes FATP1 protein, a transporter that promotes FFA uptake into cells [9]. To investigate whether human tubular epithelium also increases *SLC27A1* mRNA expression as a biological response to cold stimuli, we quantified *SLC27A1* mRNA expression in our HK2 culture model using real-time RT-PCR. *SLC27A1* mRNA expression was significantly higher at 28 °C compared with that at 37 °C (Fig. 3a). In conjunction with this phenomenon, we considered it important to simultaneously analyze the expression of PPARα, a master transcription factor for lipid metabolism, and CD36, a well-known fatty acid transporter. mRNA expression of *PPARA* and *CD36* in the hypothermia group was not significantly different from controls (Fig. 3b). Western blot analysis revealed that FATP1 protein expression in HK2 cells was elevated at 28 °C compared with that at 37 °C (Fig. 3c). Next, we performed BODIPY staining to find out whether FATP1 upregulation contributes to the formation of lipid droplets at low temperatures. HK2 cells cultured with PA under low-temperature culture conditions displayed abundant intracellular lipid droplets (Fig. 3d). In contrast, we only detected a few lipid droplets in cells cultured under other conditions (Fig. 3d). Quantification of BODIPY-positive fluorescence intensity in PA-treated HK2 cells showed that cells cultured at 28 °C exhibited significantly higher intensities compared with cells cultured at 37 °C. Furthermore, addition of the FATP1 inhibitor FATP1-in-1 significantly reduced the increase in fluorescence intensity caused by PA addition and low-temperature incubation (Fig. 3e).

### Quantitative RT-PCR analysis of renal tissue

**Fig. 4:**
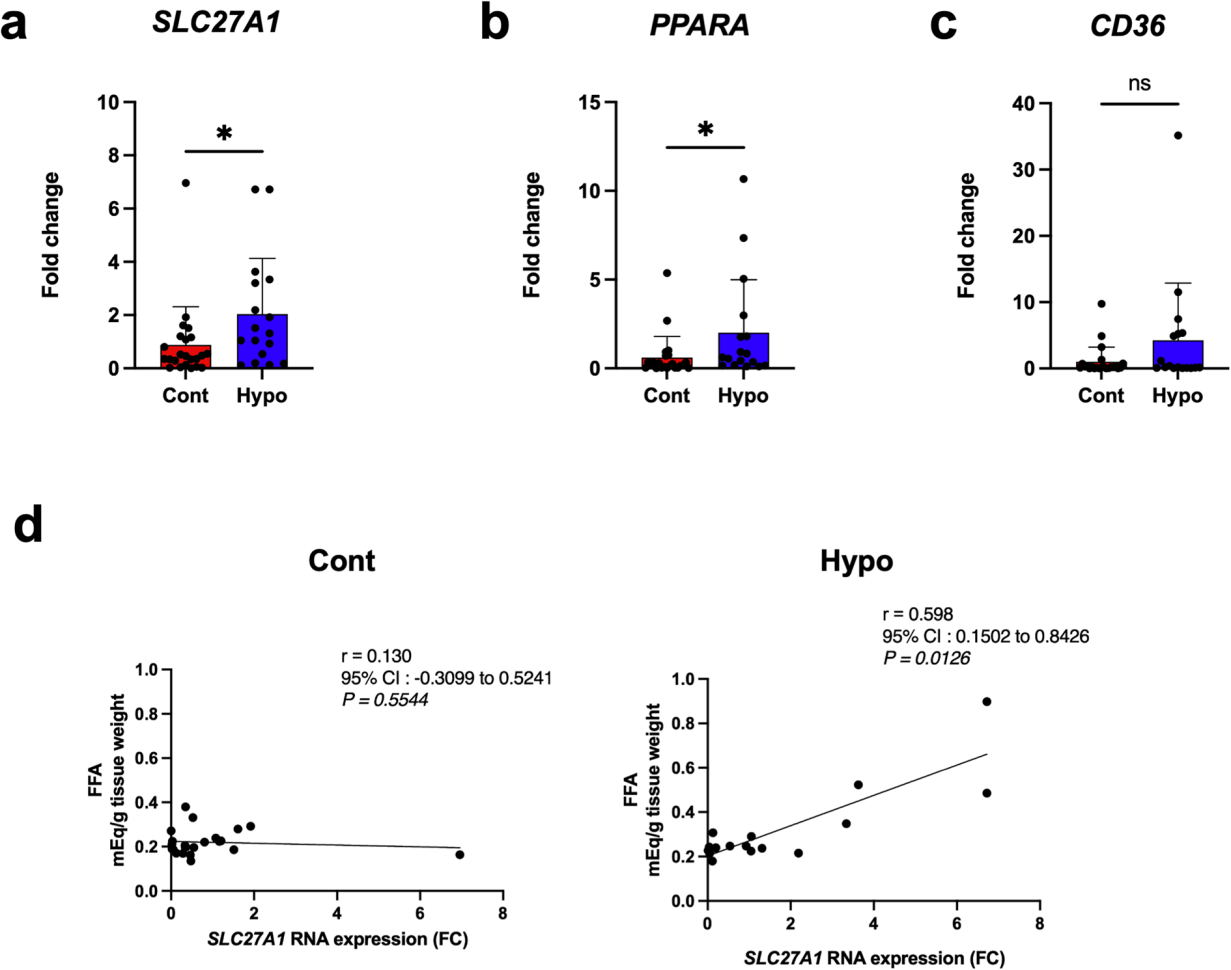
mRNA expression and correlations with lipid content in human kidney samples. (**a**–**c**) mRNA expression levels of lipid metabolism-related genes in kidney samples from control and hypothermia cases, analyzed by qPCR: (**a**) *SLC27A1*, (**b**) *PPARA*, and (**c**) *CD36* (Control: *n* = 23, Hypothermia: *n* = 17). (**d**, **e**) Correlation analysis in hypothermia cases: (**d**) *SLC27A1* mRNA expression vs. tissue FFA concentration in control (left) and hypothermia (right) cases; (**e**) *PPARA* mRNA expression vs. tissue TG concentration in hypothermia cases (*n* = 17). Data were analyzed using the Mann–Whitney U test and non-parametric Spearman’s rho correlation test. All data are represented as mean ± SEM. *P < 0.05. FFA, free fatty acid; TG, triglyceride

Based on cell culture experiments, forensic samples from hypothermia cases were expected to show changes in mRNA and protein expression associated with lipid metabolism. We assessed mRNA expression of *SLC27A1*, *PPARA*, and *CD36* in renal tissues from hypothermic forensic samples by real-time RT-PCR. *SLC27A1* and *PPARA* mRNA levels were significantly higher in hypothermia cases compared with those in controls (Fig. 4a and b). However, no significant changes were observed in *CD36* mRNA expression (Fig. 4c). *SLC27A1* mRNA expression was positively correlated with tissue FFA content in the hypothermia group but not controls (Fig. 4d).

### Protein expression of FATP1 in renal tissue

**Fig. 5:**
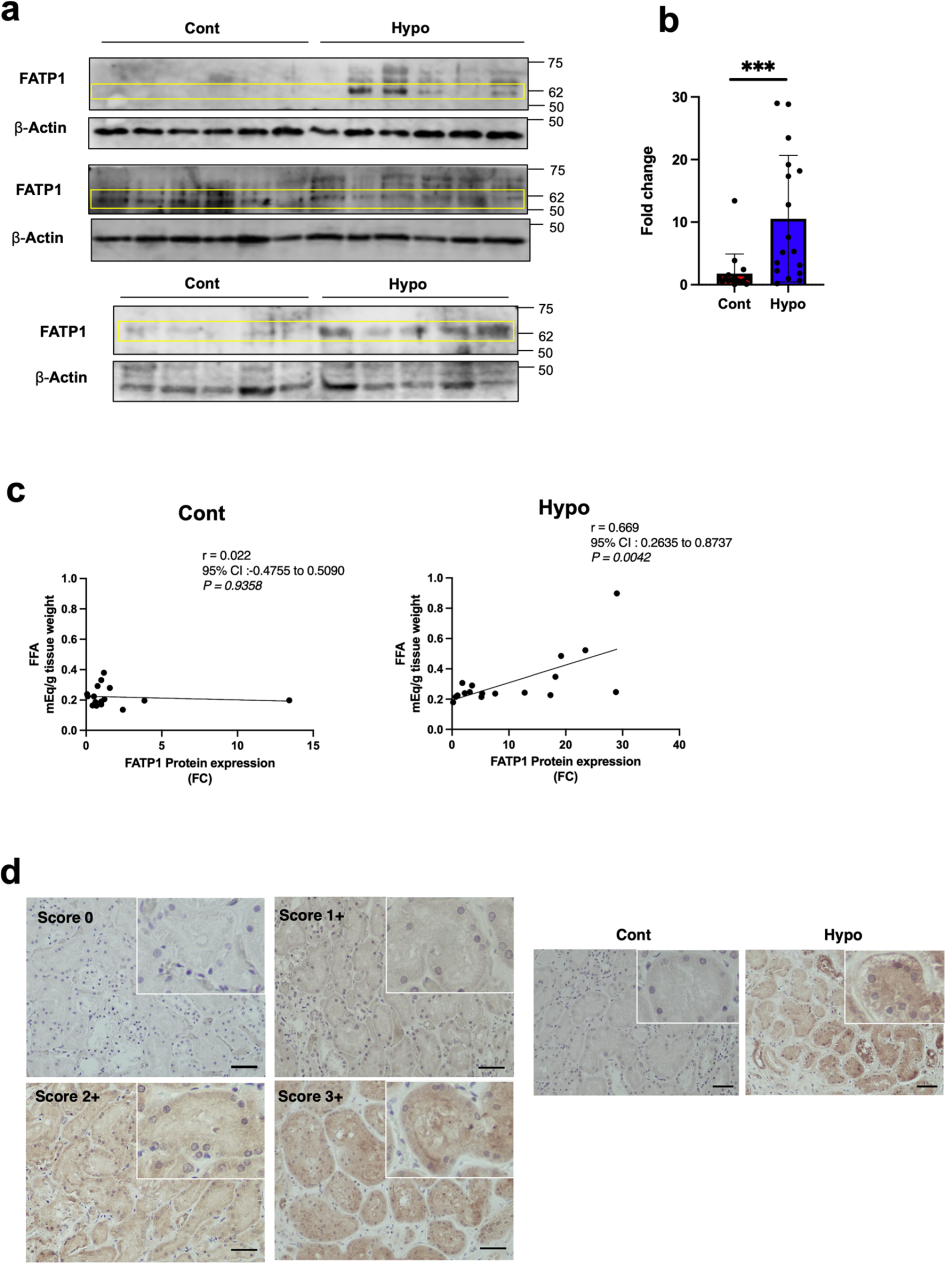
FATP1 protein expression in human kidney tissue. (**a**) Western blot analysis of FATP1 protein levels in kidney tissues from control and hypothermia cases (*n* = 17 each). (**b**) Quantitative data of FATP1 protein levels normalized to β-actin levels. (**c**) Correlations between FATP1 protein levels and tissue FFA concentrations in control (left) and hypothermia (right) cases (*n* = 17). (**d**) Immunohistochemical staining of FATP1 in renal tissues. Representative images show FATP1 localization and expression. Scale bars: 50 μm. Data were analyzed using the Mann–Whitney U test and Spearman’s rho correlation test. All results are presented as mean ± SEM. ***P < 0.001. FFA, free fatty acid

Western blot analysis revealed increased expression of FATP1 in renal tissues of hypothermia cases compared with that in tissues of control cases (Fig. 5a). Immunohistochemical analysis further showed a diffuse positive signal for FATP1 in the proximal tubular cells of hypothermic cases (Fig. 5b). FATP1 protein expression showed a significant positive correlation with tissue FFA content in hypothermia cases, while this correlation was not observed in the control group (Fig. 5c). FATP1 positivity in renal tubular cells, as assessed by immunohistochemistry, was classified into scores ranging from 0 to 3+ (Fig. 5d). Among the hypothermia group, 13 cases showed positive FATP1 scores, while 7 cases were positive in the control group (Fig. 5d; Table 3). Statistical analysis using a χ² test demonstrated that FATP1 immunohistochemistry results significantly differentiated the hypothermia group from the control group (*P* < 0.05).

**Table 3:** Positive rate of FATP1 in control and hypothermia cases

	Score				Positive rate
	0	1+	2+	3+	(%)
Control (*n* = 23)	16	3	3	1	30
Hypothermia (*n* = 17)	4	2	3	8	76

## Discussion

In response to low temperatures, humans maintain body temperature through various endogenous heat production mechanisms, including involuntary muscle contractions (shivering) and non-shivering thermogenesis [16]. During non-shivering thermogenesis, brown adipose tissue (BAT) releases FFAs as an energy source to play a crucial role in regulating body temperature [17–19]. We recently reported that metabolomic analysis of postmortem femoral blood samples from hypothermia cases showed increased energy metabolism in BAT, leading to elevated concentrations of FFAs and their metabolites in the blood [20]. In the present analysis, we first performed ORO of frozen kidney tissue to assess lipid accumulation and evaluate whether it could help in the postmortem diagnosis of hypothermia. As shown in Table 2, among the specimens available for analysis in this study, many cases of hypothermia were strongly positive for ORO, indicating that it may facilitate the postmortem diagnosis. Furthermore, in the hypothermia group, renal tissues with higher ORO grades exhibited significantly higher TG concentrations compared to the control group. These findings suggest that ORO staining reflects tissue TG levels. However, ORO staining is not a quantitative method and may be influenced by variations in tissue sampling sites. Therefore, while renal lipid deposition is a characteristic feature of hypothermia, a more reliable diagnostic approach would require quantification of lipids from tissue extracts obtained from multiple sites, rather than relying entirely on ORO staining.

Under physiological conditions, the renal proximal tubules play a crucial role in reabsorbing solutes such as sugars, amino acids, and electrolytes filtered by the glomerulus in a process that is highly ATP-dependent [6, 21]. FFAs serve as the primary energy source for proximal tubule cells, which absorb them predominantly via CD36, a fatty acid transporter expressed mainly in these cells [22]. FATP1, another transporter from the FATP/SLC27 family, also facilitates cellular FFA uptake [23]. Additionally, many lipid metabolism-related genes are regulated by the master transcription factor PPARα [8]. Wu et al. reported that FATP1 is upregulated following cold exposure, concomitant with an increase in FFA uptake in BAT by an unknown regulatory mechanism [24]. Recently, we found that hypothermia enhances FFA uptake into proximal tubular cells via FATP1, with high PPARα expression observed in both a mouse hypothermia model and cultured cells [9].

Excess FFAs may cause cellular damage, especially when accumulating in non-adipose tissues [25]. When FFAs are taken up, mitochondria rapidly use some as substrates for β-oxidation [26]. However, excess FFAs are converted into TGs, which are not harmful to the cell within lipid droplets. In other words, if FFAs are quickly converted into β-oxidation substrates or accumulated as TGs, damage to the cell is minimized. Furthermore, in a mouse hypothermia model, administration of a FATP1 inhibitor induced apoptosis in renal tubular cells, suggesting that FATP1 is the primary transporter responsible for increased FFA uptake and survival of renal tubular cells during hypothermia [9]. In this study, gene expression analysis of HK2 cells cultured at low temperatures revealed that cold exposure directly increased *SLC27A1* mRNA and FATP1 protein expression, while levels of *PPARA* and *CD36* mRNA remained unaffected. Similarly, both *SLC27A1* and *PPARA* mRNA were elevated in hypothermic renal tissue, often with positive ORO; in contrast, *CD36* mRNA showed no significant changes. These findings align with data from our prior mouse hypothermia model and experiments on mouse renal tubular cells exposed to cold. Examining correlations between mRNA expression and lipid contents, we observed a significant positive correlation between *SLC27A1* mRNA and FFA content in renal tissues of the hypothermia group, while this correlation was absent in the control group. For the hypothermia group, western blot analysis revealed abundant FATP1 protein expression, while immunohistochemistry demonstrated positive staining in approximately 75% of cases. In contrast, only minimal FATP1 protein was detected by western blot analysis in the control group, with immunohistochemistry showing a positive signal in 30% of cases. These findings suggest that FATP1 is low expressed under normal conditions, as evidenced by its slight expression [27], but its expression is significantly elevated under hypothermic conditions. Importantly, FATP1 protein levels were also positively correlated with tissue FFA levels under hypothermic conditions. These observations suggest that lipid accumulation in renal tubules originates from FFA uptake and is facilitated by increased FATP1 expression at low temperatures. This implies that low temperatures may directly trigger mechanisms that upregulate FATP1 expression. Supporting this, our findings show elevated FATP1 expression in HK2 cells cultured at low temperature, even in the absence of PA loading in the culture medium. Additionally, a low culture temperature facilitated lipid uptake by HK2 cells in the presence of PA loading, an effect that was suppressed by a FATP1 inhibitor. Therefore, in response to cold exposure, renal tubular cells may initially upregulate FATP1 to enhance FFA uptake.

While this study focuses on lipid droplet formation in renal tissue during hypothermia, it has practical limitations. In some cases of fatal hypothermia, the cooling process might be slow and hence include a long period of no intake of food or drink. In forensic autopsies, lipid droplets have been reported to form in the renal tubules of individuals with lethal ketoacidosis including starvation victims [28]. Besides, it has been reported that recurrent hypoglycemia upregulates the FATP1 expression in adipose tissue [29]. The current study was however not designed to assess the possible impact of glucose concentration on the FATP1 expression. Differences in the medical conditions in which lipids accumulate in the renal tissue from hypothermia victims remain to be investigated.

In conclusion, our analysis of forensic autopsy specimens from hypothermia cases suggests that lipid accumulation in renal tubules, alongside FATP1 upregulation, may serve as a valuable marker for diagnosing hypothermia. Consistency with findings from our previous mouse hypothermia model indicate that this phenomenon is conserved across mammals. Moreover, it implies that FFA uptake via FATP1 plays a crucial role in the body’s resistance to hypothermia, offering new insights into physiological responses to cold exposure.
